# A single-site randomized controlled trial of partner navigation to HCV treatment for people who inject drugs: a study protocol for the You’re Empowered for Treatment Initiation (YETI) partner trial

**DOI:** 10.1186/s13063-024-08662-0

**Published:** 2025-01-22

**Authors:** Meghan D. Morris, Judy Y. Tan, Claire C. McDonell, Maia Scarpetta, Tiffany N. Nguyen, Jennifer C. Price, Torsten B. Neilands

**Affiliations:** 1 Department of Epidemiology & Biostatistics, University of California, San Francisco, USA; 2 Cancer Research Center for Health Equity, Division of Population Sciences, Department of Biomedical Sciences, Cedars-Sinai Medical Center, Los Angeles, USA; 3 Department of Medicine, University of California, San Francisco, USA; 4 Center for AIDS Prevention Sciences, Division of Prevention Science, University of California, San Francisco, USA

**Keywords:** Hepatitis C, HCV, HCV treatment, People who inject drugs, Dyad intervention, Partner support, Clinical trial, Randomized trial

## Abstract

**Background:**

Disparities persist in testing and treatment for hepatitis C virus (HCV), leaving socially marginalized populations, including people who inject drugs (PWID), less likely to benefit from curative treatment. Linkage services are often insufficient to overcome barriers to navigating the medical system and contextual factors.

**Methods:**

The You’re Empowered for Treatment Initiation (YETI) Partner trial is a single-site randomized controlled trial evaluating the efficacy of a two-session behavioral intervention that engages injecting partners as peer navigators for HCV treatment. We aim to recruit 250 PWID and their primary injecting partners in San Francisco, California, randomizing them 1:1 to either a control or intervention group. The primary outcome is the initiation of HCV treatment, with secondary outcomes including treatment completion and sustained virologic response 12 weeks post-treatment. Data will be collected through questionnaires and electronic health records and analyzed using intention-to-treat and mixed-effects models.

**Discussion:**

This trial will provide evidence of a new HCV treatment linkage intervention leveraging the support of primary injecting partners to initiate HCV treatment. If successful, the intervention could inform public health strategies and policies to address HCV in marginalized populations.

**Trial registration:**

ClinicalTrials.gov NCT06179498. Registered on December 22, 2023.

**Supplementary Information:**

The online version contains supplementary material available at 10.1186/s13063-024-08662-0.

## Administrative information

Note: the numbers in curly brackets in this protocol refer to SPIRIT checklist item numbers. The order of the items has been modified to group similar items (see http://www.equator-network.org/reporting-guidelines/spirit-2013-statement-defining-standard-protocol-items-for-clinical-trials/).

**Table:** 

Title {1}	You’re Empowered for Treatment Initiation (YETI) Partner Study: study protocol for a single-site randomized controlled trial of partner navigation to HCV treatment for people who inject drugs
Trial registration {2a and 2b}.	NCT06179498
Protocol version {3}	Protocol version 1.4
Funding {4}	NIH NIDA R01DA0555325.
Author details {5a}	Meghan D. Morris, MPH, PhD Department of Epidemiology, School of Medicine, University of California, San Francisco Email: meghan.morris@ucsf.edu Judy Y. Tan, PhD Cancer Research on Diversity, Equity, and Inclusion, Cancer Center, Cedars Sinai Email: judy.tan@cshs.org Claire C. McDonell, MS Department of Epidemiology, School of Medicine, University of California, San Francisco Email: claire.mcdonell@ucsf.edu Tiffany N. Nguyen, MPH Department of Epidemiology, School of Medicine, University of California, San Francisco Email: tiffany.nguyen5@ucsf.edu Jennifer Price, MD, PhD Department of Medicine, School of Medicine, University of California, San Francisco Email: jennifer.price@ucsf.edu Torsten B. Neilands, PhD Center for AIDS Prevention Sciences, Division of Prevention Science, University of California, San Francisco Email: torsten.neilands@ucsf.edu
Name and contact information for the trial sponsor {5b}	Tamara Haegerich, PhD, Program Officer tamara.haegerich@nih.gov
Role of sponsor {5c}	The National Institute on Drug Abuse (NIDA), the study sponsor, is not directly involved in study design, data collection and management, analysis or writing of reports. They will not have any role in submission of publications. NIDA does periodically review summary reports for progress.

## Introduction

### Background and rationale {6a}

In the USA, approximately 45–60% of PWID are estimated to have HCV infection, compared to approximately 1% of the general population [1, 2]. Adults under 30 years of age experience the highest rates of incident HCV infection [3–5]. The recent opioid epidemic has contributed to an increase in young adults injecting drugs [6–8]. In San Francisco, PWID in the last 12 months accounted for only 2.8% of the total population, yet 73.1% of people with anti-HCV antibodies and 90.4% of untreated, active HCV infections in 2019 were among PWID [9]. Given this disproportionate impact, local, national, and global organizations have recognized PWID as a priority population for HCV treatment [10].

Extreme marginalization results in severe hardships for PWID, including poverty, homelessness, and discrimination, leading to distrust of and disconnection from medical systems [11–13]. PWID also typically lack traditional social support networks. Most younger adult PWID inject daily within a social network, establishing close relationships with members of their injecting network. Often, one person assumes the role of the “primary injecting partner,” providing emotional and social support that promotes well-being [14]. Studies demonstrate that having a friend or partner concerned about one’s HCV is significantly associated with treatment initiation [15], and positive concordant couples (i.e., dyads where both partners are living with untreated HCV) often consider sequentially treatment to ensure mutual support [16].

Once HCV treatment has commenced, adherence rates among PWID and former PWID are high [17–21], highlighting the importance of treatment initiation. In 2015, our research group published one of the first papers differentiating primary injecting partnerships from other social connections within injecting networks [14]. We found that primary injecting partners—typically the main person with whom drugs were injected—invested more emotional and monetary resources into the primary injecting relationship compared to other relationships. Partnerships provide physical assistance and positive emotional and social support, improving well-being in the context of marginalization [22–26].

Dyad interventions have been effective in improving health and well-being across various health areas [27–30]. Findings suggest that primary injecting partnerships are an innate resource to be leveraged in interventions aiming to improve health behaviors among PWID [12, 22, 31–33]. Thus, the existing connection between injecting partners offers a promising avenue for supporting PWID in initiating HCV treatment.

Our study, the You’re Empowered for Treatment Initiation (YETI) Partner Study, evaluates a dyad intervention that augments the social support role of one’s primary injecting partner to improve the probability of and reduce the time to initiating treatment among PWID in San Francisco, California.

### Objectives {7}

The primary objective of the study is to estimate the proportion of people newly diagnosed with chronic HCV infection who start direct-acting antiviral (DAA) treatment and the time from enrolment to starting DAA treatment by a randomized group.

H1a: Those randomized to the intervention will have a higher 6-month probability of treatment initiation compared to those in the control group.

H1b: Those randomized to the intervention will have a significantly shorter time to HCV treatment initiation compared to those in the control group.

Secondary objectives are:

- To measure changes in injecting-related interpersonal factors and partner support factors at 1-week, 1-month, 4-month, and 7-month post-randomization by randomized group.
- To determine between-group differences in HCV DAA treatment completion and sustained virologic response at 12 weeks post-treatment completion.

#### Trial design {8}

The *Y*ou’re *E*mpowered for *T*reatment *I*nitiation (YETI) Partner study is a non-blinded, parallel-group, two-arm, superiority randomized controlled trial with 1:1 allocation ratio to assess the efficacy of a two-session dyadic intervention to involve injecting partners as peer navigators to HCV treatment.

**Fig. 1:**
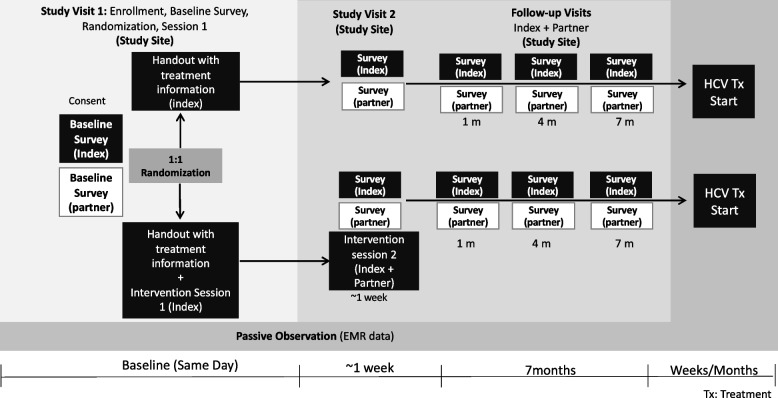
YETI Partner Study design

**Fig. 2:**
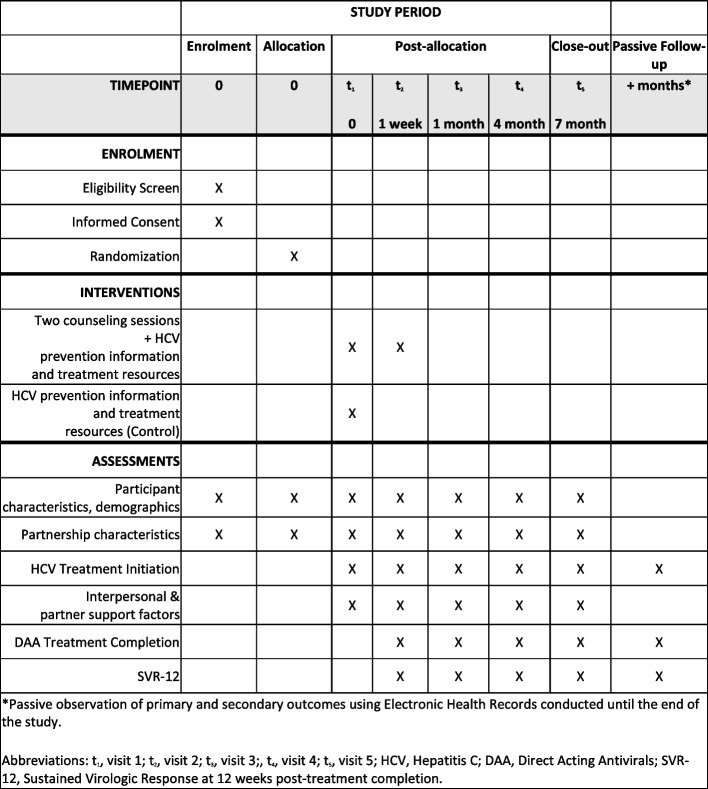
YETI Partner Study schedule of enrollment, inventions, and assessments

We aim to recruit and randomly allocate 250 persons living with HCV (“Index participants”) and their primary injecting partners (“Partner participants”) to either a control or intervention group. The primary endpoint is starting HCV treatment; secondary endpoints are treatment completion and sustained virologic response at 12 weeks post-treatment completion (sustained virologic response 12 weeks post-treatment, SVR-12). A description of the overall study design is in Fig. 1 and the schedule of enrollment, interventions, and assessments is in Fig. 2. Eligible PWID with evidence of HCV infection who enroll will be randomized (1:1) to either a control or intervention group. Both Index and Partner participants will participate in all five study visits: baseline (visit 1), 1-week (visit 2), 1-month (visit 3), 4-month (visit 4), and 7-month (visit 5). Questionnaires will be administered to the Index and Partner at all five visits. Intervention session 1 will be administered with the Index participant only at visit 1, and intervention session 2 with both the Index and Partner participants at visit 2. To expand our ability to capture primary endpoint data, participant data will be linked to their electronic health record (EHR) for HCV treatment start date, completion date, and SVR-12. The study protocol (version 1.4, 03/21/2024) follows the Standard Protocol Items: Recommendations for Interventional Trials (SPIRIT) Statement. The trial has been registered at ClinicalTrials.gov (ID: NCT06179498).

## Methods: participants, interventions, and outcomes

### Study setting {9}

The trial will be conducted in San Francisco, CA, USA. Community-based organizations (CBO) and clinics providing services for PWID will perform HCV diagnostic testing and refer eligible clients. Recruitment sites serve a set of patients diverse in racial and ethnic identities and are in neighborhoods across San Francisco Study activities will take place at a single study site located in the city center close to several CBO sites, multiple transit lines, and social service organizations that serve the focal population. The study site provides private spaces to conduct research activities, secure locations for participants to store their belongings, and food and harm reduction supplies.

### Eligibility criteria {10}

#### Eligibility

PWID with recently diagnosed HCV and their primary injecting partner will be eligible for the study.

Index participant eligibility criteria:

1. 18 years or older of age;
2. Self-report injecting drugs within the past month;
3. Have evidence of chronic HCV infection (antibody and RNA reactive) and have been diagnosed after 2016;
4. Have no evidence of HCV treatment initiation, specifically DAA treatment;
5. Self-report having a primary injecting partner whom they are willing to invite to participate in the study;
6. Cognitive capability to provide written informed consent; and
7. English language proficient.

Partner participant eligibility criteria:

1. Identified and invited to participate by the Index participant;
2. 18 years or older of age;
3. Cognitive capability to provide written informed consent; and
4. English language proficient.

Index participant exclusion criteria:

1. Previous participation in this research study (i.e., Partner participants are unable to enroll as Index participants)
2. Under 18 years of age at enrollment
3. Intention to move outside of San Francisco in the next 6 months

Partner participant exclusion criteria:

1. Previous participation in this research study (i.e., Index participants are unable to enroll as Partner participants)
2. Under 18 years of age at enrollment

#### Eligibility screening process

When a referred client contacts the YETI Partner study, staff will confirm study eligibility, collect contact information, and schedule a study visit. Referred clients will be able to contact the YETI Partner study via telephone, email, and in person at the study site.

### Who will take informed consent? {26a}

#### Written consent to participate

Eligible participants (Index and Partner) who present in-person at the study site for visit 1 will individually undergo a fully informed written e-consent protocol led by trained study staff. Research participants should be able to understand, as completely as possible, what procedures, risks, benefits, alternatives, and rights are involved, and make an informed choice about being in the study without pressure or undue inducement to participate.

During this process, consent will be requested for (a) study participation and (b) the release of medical records for up to 5 years post-participation. To help protect our participants’ privacy, we have received a Certificate of Confidentiality from the National Institute of Health. Our consent form and plain language companion document, approved by the IRB at our university, were developed in partnership with members of our community-academic partnership through a series of group discussions focused on improving readability and transparency. Directly after voluntary consent is obtained and documented on REDCap, participants will begin visit 1. REDCap (Research Electronic Data Capture) is a secure, web-based software platform designed to support data capture for research studies, providing (1) an intuitive interface for validated data capture; (2) audit trails for tracking data manipulation and export procedures; (3) automated export procedures for seamless data downloads to common statistical packages; and (4) procedures for data integration and interoperability with external sources [35, 36]. Study data were collected and managed using REDCap electronic data capture tools. Those who decline study enrollment will receive information about HCV prevention strategies and HCV treatment resources and will be paid $5 for their time.

### Additional consent provisions for collection and use of participant data and biological specimens {26b}

This trial does not involve the collection or storage of biological specimens. As such, there are no provisions or protocols for their handling within this study.

## Interventions

### Explanation for the choice of comparators {6b}

The comparator—control arm—is information about HCV prevention strategies and HCV treatment resources. In partnership with CBOs and clinics providing HCV testing and results disclosure, we will use existing materials from organizations that provide HCV testing. These materials will provide details about prevention and care which includes (a) preventing further harm to their liver, (b) reducing risks for transmitting HCV to others, and (c) undergoing medical evaluation for liver disease and possible treatment. Standard of care information focuses on information and transmission risk reduction for the individual.

### Intervention description {11a}

In addition to the standard of care HCV prevention and care handout that the control arm receives, the intervention arm participants will receive two counseling sessions (see Table 1). The intervention is manualized, with session 1 involving the Index participant only, and session 2 involving both Index and Partner participants. Each session will be led by a study staff member who is a certified HIV/HCV test counselor in California and has received additional training in motivational interviewing. Staff are trained in using the HCV Treatment Navigation Map (Navigation Map). The Navigation Map is a printed, fillable “map” comprising self-identified priorities, the steps that the Index participant will identify toward HCV treatment initiation, and the support that the Index participant will identify needing from their primary injecting partner towards treatment initiation.

**Table 1:** Intervention session information

Intervention session	Timepoint	Participant(s)	Focus
1	Visit 1	Index only	(a) Set HCV treatment as a priority goal (b) Outline Navigation Map
2	Visit 2	Index and partner	(a) Engage Partner in Index’s HCV treatment initiation plan (b) Complete the Navigation Map with Partner

#### Session 1

Index participants will receive a ~ 30-min, in-person, one-on-one counseling session led by study staff to begin filling in their individual Navigation Map (see “Protocol”). During this process, the staff member will strategically engage the participant in a series of open-ended questions that correspond to consecutive sections of the Navigation Map to build dyadic capacity for supporting the Index to start HCV treatment. Dyadic capacity depends on interpersonal factors (e.g., intimacy) and partner involvement (e.g., tangible support). Session 1 focuses on the Index participant, their situational challenges, and their individual and relational strategies or supports for treatment initiation. Study staff will use discussion probes to cultivate dialogue about past situations where the Index participant and their partner provided support (partner involvement) for other priority activities (e.g., accessing harm reduction materials or securing housing), and the strategies they employed to overcome perceived obstacles in similar situations. The study staff will engage the recently diagnosed Index participant to (a) assess HCV treatment as a priority goal, (b) outline the “HCV Treatment Navigation Map” with potential situational and contextual challenges and strategies and align priorities with motivation for HCV treatment, and (c) brainstorm how their Partner participant can support their treatment initiation.

#### Session 2

Session 2 builds on the progress made in increasing overall partner support and emphasizing the Partner’s role in tailoring support for the Index to begin HCV treatment. Study staff will facilitate a 40–60-min, in-person discussion-based activity with the Index and Partner participants that builds upon the Session 1 information. First, the research staff will assist the Index in sharing the Navigation Map from session 1 to review perceived barriers and facilitators to HCV treatment initiation. Session 2 will focus on (a) collaboratively reviewing the Navigation Map with the Index and Partner, (b) outlining and discussing preliminary strategies and timeline towards treatment initiation, and (c) role-playing and practicing strategies to concretize a plan involving both partners. Study staff will then facilitate a discussion between the Index and Partner to collaboratively devise ideas and strategies to overcome each barrier, drawing upon support strategies identified earlier (partner involvement). The Partner will also have an opportunity to fill out their own Navigation Map, highlighting ways that they can support the Index. By the end of session 2, the Index participant’s Navigation Map will list possible partner involvement strategies layered on top of challenges identified by the Index participant during Session 1. If time permits, the Index and Partner will practice applying one or two strategies to a hypothetical situation to gain confidence and familiarity.

In situations where the Partner is unable to attend session 2, facilitation will be adapted to focus on Index-only perspectives about past partner involvement and support to overcome challenges. The study staff will use the “Empty-Chair technique” whereby the staff facilitates session 2 activities based on the Index participant’s perceptions of how the Partner participant will respond in preparing the Index participant to engage the Partner for support in navigating HCV treatment. The Index participant will leave with a completed Partner Navigation Map and prompts to introduce the strategies to their partner.

### Criteria for discontinuing or modifying allocated interventions {11b}

There will be no special criteria for discontinuing or modifying allocated interventions, other than participants voluntarily withdrawing from the study or being lost to follow-up.

### Strategies to improve adherence to interventions {11c}

The intervention will be delivered in two in-person counseling sessions held 1 week apart. Adherence in this context refers to attending and completing the two planned study sessions. To support this objective, we will incorporate several strategies rooted in our experience working with PWID and conducting dyadic studies to maximize participant retention.

- Outreach: Using regularly updated contact information, study outreach workers will maintain participant contact via e-mail, social media, text messaging, phone calls, and visiting frequented physical locations.
- Incentives: Cash payments will follow an incentive structure suggested by our community-academic partnership.
- Relationships with Community Collaborators: To expand study outreach and assist with locating, we will provide CBO partners monthly with a list of participants with outstanding visits.

### Relevant concomitant care permitted or prohibited during the trial {11d}

Implementation of the intervention will not require alterations to the usual care pathways—standard of care HCV prevention and care handouts—and these will continue for both trial arms. The study will not interfere with or restrict participants’ access to usual care, ensuring that all standard services remain available throughout the trial.

### Provisions for post-trial care {30}

There is no anticipated harm for trial participation, and participants will be compensated for their time and involvement in the study.”

### Outcomes {12}

The primary outcome of this trial will be the initiation of HCV treatment within the study period. Secondary outcomes will include (a) completion of HCV treatment; and (b) achievement of SVR12. All outcomes will be measured within 2 years of enrollment. Individuals without evidence of an HCV-associated visit or without indication of DAA treatment prescribed will be considered to have not started HCV treatment. Outcomes will be assessed using data from questionnaire responses and EHR integration.

### Participant timeline {13}

#### Follow-up study visits

Irrespective of the randomized group, both Index and Partner participants individually will complete in-person questionnaires administered by study staff on interpersonal factors and partner involvement, outcomes, and illicit drug use and service usage at 1-week (visit 2), 1-month (visit 3), 4-month (visit 4), and 7-month (visit 5). In both study arms, Index participants are provided with $50 cash reimbursements after each completed visit. If the Partner attends and completes the study visits with Index participants, each will receive a $55 cash reimbursement after each completed visit.

### Sample size {14}

The primary superiority hypothesis for this trial, that those randomized to the intervention will have a higher 6-month probability of treatment initiation compared to those in the control group, was powered based on our previous work and comparable results of community-based RCT studies with PWID that suggest a HCV treatment initiation rate of 30% for those in the intervention group (13% in control group), and a retention rate of 80% (based on our previous cohort of injecting partnerships) [37, 38]. We will have 80% power to detect this effect with a sample size of 250, corresponding to a risk ratio of 2.0, at the 5% significance level.

### Recruitment {15}

Participants will be recruited through referrals (e.g., phone calls and in-person study handouts to clients) from CBOs and clinics providing HCV testing. Study staff will also perform street outreach, passing out study handouts to interested people around San Francisco venues where PWID frequent.

## Assignment of interventions: allocation

### Sequence generation {16a}

The sequence of random assignments will be generated and uploaded into REDCap for use with enrollment. A simple randomization allocation table will be generated using sorted random values to distribute allocation to the intervention or control arm. This will allow for concealment allocation as research staff will not be able to anticipate allocation.

### Concealment mechanism {16b}

Group assignment of participants is carried out by a random assignment tool within REDCap. The randomization allocation table and settings will only be accessed by the data manager.

### Implementation {16c}

Participants will be enrolled by the study staff at primary study sites. The assignment of participants to either the intervention or control arm will be done electronically through REDCap at the time of randomization. Participants cannot be reassigned to different arms.

### Assignment of interventions: blinding

#### Who will be blinded {17a}

Due to the nature of the study design, the delivery of the intervention will not be blinded. The result of the concealment of a new step in the allocation sequence will be disclosed to participants and staff. Those involved in the data analyses will be blinded to the group allocation. All analyses will be performed by the study’s biostatistician, select study staff under the guidance of the study’s biostatistician, and the principal investigators (PIs) while blinded to study allocation.

#### Procedure for unblinding if needed {17b}

The study participants and study staff interacting with participants will not be blinded. Analytic datasets will be blinded to allocation. Unblinding will occur only after all statistical analyses are completed and the primary outcomes have been fully analyzed and documented.

Unblinding of a participant’s allocated intervention will be permissible under the following circumstances: (1) medical emergency: if a participant experiences a serious adverse event or medical emergency where knowledge of the intervention is necessary for providing appropriate medical care, or (2) regulatory request: if requested by regulatory authorities for safety monitoring or audit purposes.

### Data collection and management

#### Plans for assessment and collection of outcomes {18a}

Following randomization, participants will complete detailed questionnaires at enrollment, 1 week, 1 month, 4 months, and 7 months. All analyses will be performed by the study’s biostatistician as well as by study team members and collaborators under the guidance of the study’s biostatistician and the PIs while blinded to study allocation.

#### Data sources

The YETI Partner Study will use a combination of EHR and questionnaires to collect data.

EHR data will include HCV testing, treatment initiation, completion, and cure information, which is requested every 3 months for Index participants via medical record number (MRN).

Self-report data will be collected individually through staff-administered questionnaires at the baseline screening visit, after informed consent, and all subsequent visits.Data will be collected individually and in separate rooms for the Index and Partner participants to ensure accurate responses. Questionnaires will assess the following domains:

- Demographic characteristics will include age, gender, sexual orientation, race/ethnicity, education level, employment status, sources of income, food security using the USDA Household Food Security Survey Model, and living arrangements.
- Healthcare access characteristics will include access to providers, emergency care, and barriers to access.
- HCV knowledge will be measured using an adapted HCV Knowledge questionnaire from the Canada Treatment Information Exchange (CATIE).
- Drug use questions will cover methods, frequency, and substances used, equipment sharing and harm reduction practices, attitudes towards current use, receipt of opioid substitution therapy, overdose, and alcohol and tobacco use. These measurements have been adapted from questions employed by the PIs.
- Partner and relationship characteristics will be measured using information about partner dynamics, social networks, injecting dynamics, and partner support via the 54-item IDIP scale [32], the Source-Specific SPS-24 [39], and the SPS-24 adapted for HCV treatment initiation.
- Police interactions will include interactions with police and history of incarceration or parole.
- General health includes HCV history, quality of life, and depression. Depression will be measured using the standardized Center for Epidemiologic Studies Depression Scale (CESD) [40].
- Stigma and racism will be captured using the Substance Use Stigma Mechanisms Scale (SU-SMS) [41] with adaptations and the Experiences of Discrimination scale with minor adaptations in language [42].

Subsequent visits will include questions related to the Index’s and Partner’s experiences with the intervention and self-reported outcome data.

#### Plans to promote participant retention and complete follow-up {18b}

See {11c}.

The study incorporates several strategies rooted in our experience working with PWID and conducting dyad studies to maximize participant retention. Subsequent visits will be scheduled immediately after completion of the current visit. Using regularly updated contact information, study outreach workers will maintain participant contact via e-mail, text messaging, phone calls, and visiting frequented street locations. Participants will receive cash payments on a flat scale at each visit. However, dyads will be provided a higher cash incentive if they present for a visit at the same time. To expand study outreach, we will provide CBO partners with a list of participants with outstanding visits monthly.

#### Data management {19}

The YETI Partner Study will employ standardized procedures for data entry, coding, security, storage, and analysis to ensure data quality and confidentiality. Data will be entered electronically into REDCap, a secure and UCSF-approved database. The database will include close-response entries and range restrictions to maximize valid data entry. Critical data points will undergo double data entry by different team members, with discrepancies resolved through a third review at regular intervals. Automated range checks will ensure data values fall within expected ranges. Data will be entered in real-time during participant visits to reduce recall bias.

Each participant and dyad will be assigned a unique study ID number to anonymize data. Data will be coded using standardized protocols, including demographic information, health status, and questionnaire responses. All electronic data will be stored on secure, password-protected servers with restricted access, maintained by UCSF’s IT department. Physical documents will be stored in locked filing cabinets accessible only to authorized study personnel and shredded after digitization. Data linking participant identities to their study IDs will be kept in a separate, secure file with limited access. All study personnel will undergo mandatory HIPAA and privacy training.

Data will be transferred into RStudio or STATA and cleaned before analysis using the most current version. The dataset will be locked, and all analyses will be performed by the study’s biostatistician as well as study team members, and collaborators in conjunction with guidance from the biostatistician and principal investigators (PIs) while blinded to study allocation. Regular audits of data quality and completeness will be conducted monthly by a research assistant and reviewed quarterly by the PIs. Compliance with data management protocols will be ensured through regular training sessions and detailed documentation. Further details of the data management procedures are available in the study protocol.

#### Confidentiality {27}

All collected information will be kept strictly confidential and stored in accordance with national regulatory approvals. The PIs and biostatistician will be the only study personnel with access to the code list.

#### Plans for collection, laboratory evaluation and storage of biological specimens for genetic or molecular analysis in this trial/future use {33}

See above 26b—this trial does not involve the collection or storage of biological specimens. As such, there are no provisions or protocols for their handling within this study.

## Statistical methods

### Statistical methods for primary and secondary outcomes {20a}

A detailed plan for analysis will be developed before data extraction, but some of the principles of data analysis are outlined here. Analysis will be reported in accordance with the Consolidated Standards of Reporting Trails (CONSORT) statement [43]. All analyses will be performed by the study’s biostatistician as well as by select team members under the guidance of the study’s biostatistician and the PIs. All trial data will be summarized by treatment group.

Randomization should result in a balance on covariates across the intervention and control groups. In the unlikely event that imbalance is detected across groups on one or more of the demographic variables, we will use methods based on the Rubin causal model (e.g., propensity scores, double-robust estimation) to obtain intervention effect estimates under the counterfactual assumption of balanced groups.

Our primary analysis will follow an intention-to-treat (ITT) approach with no account taken of protocol non-adherence. We will address incomplete data via direct maximum likelihood estimation (MLE) or multiple imputation (MI), which make the relatively mild assumption that incomplete data arise from a conditionally random (MAR) mechanism. Auxiliary variables will be included to help meet the MAR assumption and sensitivity analyses will be conducted with controlled MI or other relevant methods to assess the robustness of the MAR assumption [44]. Well-tested commands and procedures in the statistical program R or STATA will be used to perform the descriptive and mixed models analyses planned to address questions of the mechanism of action. The specialized latent variable modeling program M*plus* will be used to fit secondary exploratory mediation and moderation analyses. All program code will be documented extensively to enable future code review, transparency, and results reproducibility.

Given the randomized design, the primary comparison will include the cumulative incidence and risk ratios with 95% CI for HCV treatment initiation by Index participants at 6 months post-enrollment and across the entire follow-up period by randomization group. We will use a survival framework that acknowledges loss to follow-up with at-risk time beginning at study enrollment and censoring at the date of the final attended follow-up visit. The event of interest is HCV treatment initiation using a known or self-reported date of HCV treatment initiation.

Secondary analyses will include comparisons of other treatment completion and SVR12 by randomization group, with subgroup analyses for a stratum of Index ≤ 30 years of age, gender, race, and ethnicity.

While all Index participants will be living with untreated HCV at the time of study enrollment, some injecting partners will also have untreated HCV infection and may benefit from intervention involvement. Exploratory analyses will measure differences in the probability of initiating HCV treatment for HCV-infected partners by randomized group.

An exploratory linear mixed model (LMM) will assess the putative mechanisms of action by which changes in partner support and interpersonal factors (over time and by group) impact HCV treatment initiation.

Additional secondary exploratory analyses with intact dyads where both partners have data on the same independent variables will enable the investigation of partnership-based research questions that explore how relationship dynamics affect behavior change in partnerships. Therefore, we will extend the mixed models described above to include actor and partner effects for the continuous partner support variables. This technique illuminates the effects that partners in injecting partnerships can have on both their own and their partner’s behavior. Estimation of separate actor and partner effects will enable us to use contrasts to test whether partner effects are greater in magnitude than actor effects. α will be set at 0.05 for these exploratory analyses.

### Interim analyses {21b}

Interim analyses will be conducted every 6 months. The trial may be stopped early for ethical reasons under three circumstances: if there is evidence suggesting that the intervention is harming participant safety. The final decision to terminate the trial will be made by the principal investigator and the study sponsor.

### Methods for additional analyses (e.g., subgroup analyses) {20b}

Subgroup analyses will be performed to examine differences in outcomes across various participant characteristics, such as age, gender, and baseline HCV status. Adjusted analyses will account for potential confounders and covariates, such as socioeconomic status, healthcare access, and injecting network size.

To address specific hypotheses, LMMs and generalized linear mixed models (GLMMs) will be employed. LMMs will be used to assess continuous outcomes, such as interpersonal factors and partner involvement levels, with repeated measures for each participant within dyads. GLMMs will be used to evaluate binary outcomes, such as HCV treatment initiation, incorporating random intercepts to account for clustering within dyads. Exploratory analyses will include mediation and moderation analyses to investigate the mechanisms through which the intervention affects outcomes and to identify factors that may influence the strength or direction of these effects.

### Methods in analysis to handle protocol non-adherence and any statistical methods to handle missing data {20c}

The primary analysis population will follow an intention-to-treat (ITT) approach, including all participants as originally assigned to their intervention groups regardless of adherence to the protocol. For protocol non-adherence, the analysis will use as-randomized analysis to maintain the validity of the randomized controlled trial design.

We will address incomplete data via direct maximum likelihood estimation (MLE) or multiple imputation (MI), which make the relatively mild assumption that incomplete data arise from a conditionally random (MAR) mechanism. Auxiliary variables will be included to help meet the MAR assumption and sensitivity analyses will be conducted with controlled MI to assess the robustness of the MAR assumption.

### Plans to give access to the full protocol, participant-level data and statistical code {31c}

The de-identified datasets analyzed during the current study and statistical code are available from the corresponding author on reasonable request, as is the full protocol. These plans are outlined in detail in our Data Sharing and Management Plan. We also plan to deposit the data and code in an open data repository, such as Zenodo or the Open Science Framework, following trial completion.”

### Oversight and monitoring

#### Composition of the coordinating center and trial steering committee {5d}

While this study does not include a formal Trial Steering Committee (TSC), oversight is ensured through regular meetings and structured processes involving the following groups:

Principal Investigator and Research Team: The PI and core team (clinical research coordinators, graduate student researchers, and data manager) provide day-to-day support. Responsibilities include participant recruitment, obtaining informed consent, intervention delivery, and data collection. The team also supports protocol development, study design and conduct, and manuscript preparation with guidance from the investigators. The team meets twice weekly with the PI to discuss trial operations and every 1–2 months to review progress, publications, and adherence to the protocol.

Community Academic Board (CAB): The CAB serves as the Stakeholder and Public Involvement Group (SPIG), composed of community representatives, patient advocates, and academic members. This board meets 6–12 times per year in person to provide input on recruitment strategies, community engagement, and dissemination plans.

Institutional Review Board (IRB): The IRB reviews the trial annually or as needed for protocol amendments, ensuring compliance with ethical and regulatory standards.

#### Composition of the data monitoring committee, its role and reporting structure {21a}

In accordance with the National Institutes of Health (NIH) and the primary funding agency, the National Institute on Drug Abuse (NIDA), we have developed a Data and Safety Monitoring (DSM) plan, and the PIs and core research team will maintain appropriate oversight and monitoring of the trial’s conduct in its entirety. As this trial is not a phase 3 trial and it was determined to be minimal risk to participants, no Data Safety Monitoring Board (DSMB) was required. The DSM plan was approved by the University of California, San Francisco Institutional Review Board (IRB) and NIDA before initiating enrollment.

#### Adverse event reporting and harms {22}

The primary risks to participants in the trial will be a breach of confidentiality and social stigma.

#### Frequency and plans for auditing trial conduct {23}

Trial conduct is monitored through internal audits conducted by the data manager on a weekly and monthly basis to ensure data quality, integrity, and adherence to the protocol. Findings are reported during biweekly operational meetings with the investigators and coordinating team. Issues occurring at the study site are reported immediately to the principal investigator and discussed during these regular meetings. Additionally, the principal investigator and coordinating team meet twice a year to specifically review the integrity of intervention delivery and adherence to the trial protocol.

#### Plans for communicating important protocol amendments to relevant parties (e.g. trial participants, ethical committees) {25}

Through March 21, 2024, three protocol amendments have occurred. Any future changes to the protocol will be reported to and approved by the UCSF Institutional Review Board (IRB) prior to implementation. Additionally, all amendments will be communicated to the study sponsor, the NIH, in accordance with reporting requirements outlined in the Data and Safety Monitoring Plan (DSMP). Any deviations from the protocol will be fully documented using a breach report form, which will be stored electronically and reviewed by the coordinating team. Major protocol breaches will be reported to the IRB within 5–10 days of the event occurrence or awareness, in accordance with UCSF IRB reporting requirements. The protocol will also be updated in the clinical trial registry (ClinicalTrials.gov) to reflect any changes.

#### Dissemination plans {31a}

The primary results of the trial will be reported in accordance with CONSORT guidelines, published in a peer-reviewed journal, and disseminated to the community via presentation at partnering community organizations and information sheet.

## Discussion

When complete, the YETI Partner Study will represent one of the first randomized clinical trials that evaluate the efficacy of an intervention to enhance partner support for starting HCV treatment. This trial will provide data on a new linkage intervention that can be integrated with existing HCV results disclosure counseling services, navigation services, and harm reduction services for PWID. If partner navigation yields at least a 20% increase in HCV treatment initiation, it could significantly impact public health strategies for addressing HCV among PWID.

Several practical and operational challenges are anticipated in performing the study. Ensuring participant engagement and retention is critical given the transient nature of the focal population and their complex social circumstances. Strategies such as providing transportation support, flexible scheduling, and regular follow-ups will be employed. Maintaining data quality and managing missing data will involve rigorous protocols, including double data entry, range checks, and using maximum likelihood and MI methods. Protecting participant confidentiality is paramount, with measures including the use of unique study ID numbers, secure data storage, and obtaining Certificates of Confidentiality.

Operational coordination with CBOs will be essential for participant recruitment and follow-up. Building strong relationships with CBOs and training their staff on study protocols and ethical considerations will be crucial. Ethical considerations, particularly obtaining informed consent and ensuring voluntary participation, will be carefully managed. The YETI Partner Study aims to address a critical gap in HCV treatment initiation among PWID by leveraging the supportive role of primary injecting partners, potentially informing future interventions and policies aimed at improving HCV treatment outcomes in marginalized populations.

### Trial status

Study protocol 1.4, 03/21/2024; recruitment began March 2024, estimated date recruitment is complete is December 2027.

## Supplementary Information

Supplementary Material 1.

## Data Availability

The data that support the findings of this study will be available from the corresponding author upon reasonable request. Access to the data will be granted following the approval of a Data Use Agreement to ensure the confidentiality and ethical use of the participant information.

## Abbreviations

PWID: People who inject drugs
HCV: Hepatitis C virus
DAA: Direct-acting antiviral
RCT: Randomized controlled trial
CBO: Community-based organization
SVR: Sustained virologic response
IDIP: Interpersonal factors underlying injection drug use
SPS-24: Source-Specific Social Provisions Scale
CESD: Center for Epidemiologic Studies Depression Scale
SU-SMS: Substance Use Stigma Mechanisms Scale
NIH: National Institutes of Health
NIDA: National Institute on Drug Abuse
IRB: Institutional Review Board
EMR: Electronic medical record
DSMB: Data Safety Monitoring Board
DSM: Data and Safety Monitoring
MRN: Medical record number
MI: Multiple imputation
LMM: Linear mixed model
GLMM: Generalized linear mixed model
MAR: Missing at random
SPIRIT: Standard Protocol Items: Recommendations for Interventional Trials
REDCap: Research Electronic Data Capture
CONSORT: Consolidated Standards of Reporting Trials
