# Supplementary material for: A single-site randomized controlled trial of partner navigation to HCV treatment for people who inject drugs: a study protocol for the You’re Empowered for Treatment Initiation (YETI) partner trial
Source: Trials. 2025 Jan 22;26:26. doi: 10.1186/s13063-024-08662-0 (PMC11753105; doi:10.1186/s13063-024-08662-0)
Supplement: Supplementary file 1 — Supplementary Material 1. [file 13063_2024_8662_MOESM1_ESM.docx]

YETI Partner Study Protocol

**Full Title:** You’re Empowered for Treatment Initiation (YETI) Partner Study: A Randomized Trial to Test the Efficacy of a Partner Navigation Intervention for HCV Treatment among People who Inject Drugs

**Grant Number:** 5R01DA05332

**Funding Organization:** National Institute on Drug Abuse

**ClinicalTrials.gov Number:** NCT06179498

**IRB Number:** 21-35045

**IRB Institution:** University of California, San Francisco

**Principal Investigator:**

Meghan Morris, MPH, PhD

Associate Professor

Vice-Chair of Diversity, Equity, and Inclusion

Department of Epidemiology & Biostatistics

Institute for Global Health Sciences

Benioff Homelessness & Housing Initiative

University of California, San Francisco

Office: 415-574-0651

Email: Meghan.Morris@ucsf.edu

**Co-Investigators:**

Judy Tan, PhD, MA

Associate Professor

Division of Population Sciences, Department of Biomedical Sciences

Cedars-Sinai Cancer Center

Email: judy.tan@cshs.org

Tor Neilands, PhD

Professor

Division of Prevention Science

Department of Medicine

University of California, San Francisco

Email: Torsten.Neilands@ucsf.edu

Jennifer Price, MD, PhD

Professor

Department of Medicine

University of California, San Francisco

Email: Jennifer.Price@ucsf.edu

**Study Contact Information:**

Email: YETIStudy@ucsf.edu

Background

Although people who inject drugs (PWID) are disproportionately impacted by Hepatitis C (HCV) infection, current strategies to engage them into curative treatment have had limited success. This proposal will rigorously evaluate a novel intervention to connect PWID living with HCV to effective cures. Due to the experience of extreme marginalization, PWID experience severe hardships—including poverty, homelessness, and discrimination—that result in distrust and disconnection with medical systems. In addition, PWID typically lack traditional relationships, such as family, friends, and co- workers. Most young adult PWID inject daily within a social network and establish a closer relationship with one member of their injecting network who assumes the role of “primary injecting partner.” Injecting partners, especially a primary injecting partner, offer emotional and social support that promotes well-being. As such, the connection between injecting partners is an underutilized resource for health promotion, and offers a promising new avenue for PWID to initiate HCV treatment.

Study Design

**Study Design:** Randomized controlled trial

- **Population:** Adults (18+), who test positive for HCV at a partner community-based organization (CBO) in San Francisco and who report lifetime injection drugs use and a main injection partner
- **Intervention:** YETI Partner Intervention
- **Control:** Standard of care
- **Outcome:** Initiation of HCV treatment (see below for secondary outcomes)
- **Time:** 6-months post disclosure

Study Aims:

The primary objective of the study is to estimate the proportion of people newly diagnosed with chronic HCV infection who start direct-acting antiviral (DAA) treatment and the time from enrolment to starting DAA treatment by a randomized group.

**H1a:** Those randomized to the intervention will have a higher 6-month probability of treatment initiation compared to those in the control group.

**H1b:** Those randomized to the intervention will have a significantly shorter time to HCV treatment initiation compared to those in the control group.

Secondary objectives are:

- To measure changes in injecting-related interpersonal factors and partner support factors at 1-week, 1-month, 4-months, and 7-months post-randomization by randomized group.
- To determine between-group differences in HCV DAA treatment completion and sustained virologic response at 12 weeks post-treatment completion.

Sample Size

Our enrollment target is 250 young adult PWID (index) with recently identified HCV infection who report a primary injecting partner to be randomized into either control (N=125) or intervention (N=125) groups. Exclusion criteria include: previous research study participation (i.e., partners are unable to enroll as index participants and index participants are unable to enroll as partner participants); under 18 years of age at enrollment

Eligibility

Index Participant Eligibility Criteria:

- 18 years or older of age;
- Self-report injecting drugs within the past month;
- Have evidence of chronic HCV infection (antibody and RNA reactive) and have been diagnosed within the past month after 2016;
- Have no evidence of HCV treatment initiation, specifically DAA treatment;
- Self-report having a primary injecting partner whom they are willing to invite to participate in the study;
- Cognitive capability to provide written informed consent; and
- English language proficient.

Partner Participant Eligibility Criteria:

- Identified and invited to participate by the Index participant;
- 18 years or older of age;
- Cognitive capability to provide written informed consent; and
- English language proficient.

Index Participant Exclusion Criteria:

- Previous participation in this research study (i.e., Partner participants are unable to enroll as Index participants)
- Under 18 years of age at enrollment
- Intention to move outside of San Francisco in the next 6 months

Partner Participant Exclusion Criteria:

- Previous participation in this research study (i.e., Index participants are unable to enroll as Partner participants)
- Under 18 years of age at enrollment

Study Activities

1. Visit 1
   1. Informed consent
   2. Randomization
   3. Baseline questionnaire
   4. Intervention counseling session I (Index randomized into intervention arm ONLY)
2. Visit 2
   1. Intervention counseling session II (Index and partner randomized into intervention arm ONLY)
   2. Questionnaire
3. Visit 3-5
   1. Questionnaire
4. Electronic health record data extraction

PHASE 1 RECRUITMENT

### Dyad Informant Interviews

In this phase we will recruit approximately 5 young adult PWID with HCV infection and their primary injecting partner (n=10 people total) to participate in cognitive interviews on a rolling basis. Participants will be recruited by our CBO partners and via street-based recruitment. In partnership with CBO staff, our research team will review a list of potential participants reflecting regular clients of their services to identify a purposively sampled target list to participate.

### CBO Intervention Assessment

We will recruit at least one CBO staff member from each venue to review study protocols and conduct mock “test and refer” sessions.

### Community Academic Partnership

In this phase, we will form a Community Academic Partnership (CAP) consisting of 8-10 young adult PWID and people living with HCV. People with lived experience of drug use and people who provide services to people who use drugs will be recruited by our CBO partners and close collaborators (End Hep C SF).

PHASE 2 RECRUITMENT

### Recruiting Index Participants

In this phase, we will recruit 250 Index participants to participate in the RCT from local CBOs performing HCV reflex testing with the following steps.

1. Potential participant receives reactive HCV RNA test result at a CBO
2. CBO staff shares the study opportunity with the patient, using the “*PNI CBO Eligibility Script”*
3. Potential participant expresses interest in participating in YETI Partner Study
4. YETI Partner Study Staff assesses patient’s eligibility:
5. YETI Partner Staff share verbal consent sheet and study information sheet with participants and ask if they would be interested in participating in a research study
6. YETI Partner Study staff gather verbal consent to access potential participants’ HCV RNA test results and ask the clients their preferred contact information

Index Participant Initial CONTACT

Overview

Once we have a potential participant’s contact information and results, eligible and interested participants who have a positive HCV result will be contacted by a study staff member to schedule Visit 1.

Protocol

**Tone:** friendly, transparent, respectful

### Contact Methods:

All first contact attempts will be made on [DOW] between [X am/pm – X am/pm] from a designated study:

- Google phone number: 415-XXX-XXXX
  - Call and text
- Email: [YETIStudy@ucsf.edu](mailto:YETIStudy@ucsf.edu)
- Study Social Media Accounts
  - Facebook: XXXX
  - Instagram: XXXX
  - TikTok: XXXX

### Attempts:

- Staff will **make at least 3 attempts and no more than 5 attempts** over the course of two weeks to contact potential participants.
- Attempts should be made first at the time indicated on the *Consent to be contacted for future research* document and then at different times (morning, midday, evening) and with different methods (phone, social media, letter) when possible.
- Attempts should be in partnership with CBO staff when possible.

### Documentation:

- All attempts – even unsuccessful – and their outcomes should be documented in a participant’s REDCap profile so that they aren’t duplicated.
- Loss to follow-up: If unable to contact participant within 30 days (or if patient refuses communication) this should be indicated on the participant’s REDCap profile and the participant’s case will be “closed” unless they reach back out to the study team.
  - A list of individuals that are not located will be kept by the Data Manager

Scripts by Contact Methods

### Phone call/In-person:

1. Introduce yourself
   1. *Hi this is [name] from UCSF. At [CBO] you shared interest in participating in a paid research study and I am following up about that.*
2. Verify their identity
   1. *Is this [*potential participant name]?
3. Ask permission or schedule time to talk
   1. Is now a good time for a 5-minute chat?
      1. Yes -> proceed to 4
      2. No -> schedule time ensuring that YOU will be the person following up
4. Assess whether the patient is aware of their HCV test
   1. *Do you remember getting your blood drawn for HCV testing at [CBO] on [date]?*
5. Introduce study and assess interest
   1. *At that time, you expressed interest in having your results shared with you through a paid UCSF research study. Are you still interested in that?*
      1. Yes -> proceed
      2. Maybe -> *Can I answer any questions for you?*
         1. See FAQ Section for appropriate responses
      3. No -> *No problem, thanks for taking the time to speak with me. You can pick up your Hep C results from [CBO]. Call us back if you change your mind.*
6. Confirm they inject drugs with others
   1. *Our study is about social support among people who inject drugs together. Do you ever inject with other people?*
      1. *Yes -> proceed*
      2. Maybe -> *Can I answer any questions for you?*
         1. See FAQ Section for appropriate responses
      3. *No -> Okay, have you ever injected with someone in the past?*
         1. *Yes -> proceed*
         2. *No -> No problem, thanks for taking the time to speak with me. You can pick up your Hep C results from [CBO]. Call us back if you change your mind.*
7. *Confirm a main injection partner*
   1. *Is there someone who you use with that could come to the study with you?*
      1. *Yes -> proceed*
         1. *Great, they will be paid for their time as well. Even though you will need to come in at the same time, your visits will be separate. You will be paid $45 cash and they will be paid $30 cash.*
         2. *We do not need to collect their information now.*
      2. Maybe -> *Can I answer any questions for you?*
         1. See FAQ Section for appropriate responses
      3. *No -> No problem, thanks for taking the time to speak with me. You can pick up your Hep C results from [CBO]. Call us back if you change your mind.*
8. Schedule visit
   1. *Great we are open on [DOW and time]. Does coming in at [Xam/pm on DOW] work for you and your injection partner?*
9. Share transportation basics if needed.
   1. *The study site is in SoMa near Civic Center. It is next to the Downtown Street Team (DST) Office at 65 9^th^ street between Market St and Mission St. Do you know how to get there?*
      1. See transportation resources by neighborhood below.
10. Confirm preferred contact information
    1. *We usually remind folks about their appointment. What is the best way to remind you?*
11. Ask if they need support coordinating with their partner
    1. *Do you need support coordinating with [name of partner]?*
12. Thank them for their time!
    1. *Thanks, we looking forward to meeting you on [DOW]! As a reminder, we cannot start your visit until your partner is there. Please call or text if you have any questions or concerns.*

### Voicemail:

Hi, this is [name] from UCSF calling for [potential participant name]. You expressed interest at [CBO clinic] to participate in our paid research study. Give us a call or text and we can share more information with you. Our number is [study phone].

### Text Message:

Hi [potential participant name] – this is [name] from UCSF. You shared interest in participating in our paid research study when at [CBO clinic]. We are reaching out to confirm your eligibility and schedule a visit! When is a good time to call you for 5 minutes?

### Social Media Direct Message:

Hi [potential participant name] – this is [name] from UCSF. You shared interest in participating in our paid research study when at [CBO clinic]. We are reaching out to confirm your eligibility and schedule a visit! When is a good time to call you for 5 minutes?

### Letter:

Use our printed team flyer and hand write the participant name at the top and your name at the bottom

VISIT 1 PROCEDURES - INDEX

FRONT DESK

1. Check participant in (see front desk procedures)
2. Prepare “Visit 1 packet” with paperclip:
   1. Visit 1 checklist
3. Fill in participant ID and date on top right corner of checklist
4. Ask participant to wait in the waiting room for interviewer to come grab them
5. Put Visit 1 packet in designated box
6. Message group chat to notify team of a check in

FACILITATOR

### Visit Preparation

1. Review REDCap profile and keep it open for data entry starting at the consenting process.
2. Pick up and quickly review information on their Visit Checklist

### Welcome

1. Welcome participant and introduce self
   1. *Hi, my name is [name] and I am going to be with you for the duration of your appointment.*
2. Walk participant back to interview station and ask them to get comfortable
   1. *To start off, I will walk you through what to expect during this visit so that you can be sure that you would like to participate. Does that sound okay?*
3. Put up “Visit in Progress” board/sign/redcap sheet
4. Introduce visit procedures
   1. *I will now review study procedures for about 10 minutes and answer any of your questions. If you are up to participate, I will ask you to voluntarily sign the consent forms. Do you have any questions before I start?*
5. Consent
   1. Introduction: *There are two forms that I will review. The first form is this our Study Consent form. This form explains what the “YETI Study” is and why you are being asked to voluntarily participate. It’s my job to make sure you understand both those of those things. We would also like ask for your consent to view your medical record so we can see if and when you get treatment for Hep C in the future. As part of that permission, we will need you to sign a HIPAA-form, which temporarily, and for the purpose of our study only, grants us access to your protected medical information directly related to hepatitis C testing and treatment.*
   2. Study Consent “Phase 2 Index Consent”
      1. Hand participant a paper copy
      2. Read the highlighted sections out loud to the participant
      3. Ask if they have any questions
   3. HIPAA
      1. *As a reminder, this is permission for our team to view your medical records, for the purposes of the study only.*
      2. Hand participant a paper copy
      3. Read through highlighted sections out loud to participant
      4. Ask if they have any questions
6. Gather e-signatures from the participant in the designated areas on both forms
   1. Note: the HIPAA forms requests for multiple initials on the middle pages
7. Generate YETI study ID and collect partner information (REDCap)

### Administer Questionnaires

1. Follow guidance in Best Practices for Survey Administration in YETI Data Collection Guide
2. Before starting, verify that the following are present.
   1. Privacy
      1. Dyad partner not in earshot
      2. White noise machine is on
      3. Dividers are up
   2. Comfort and safety
      1. Participant is settled and ready to participate
      2. Water and snack is available
3. Tell the participant what is about to happen.
   1. *Next we will complete the questionnaire. This is expected to last 30-40 minutes.*
4. Remind of ground rules, if needed.
   1. *As a reminder, you take a break, go to the bathroom, and stop for food or water at any time.*
   2. *You can skip answering any question, but we appreciate your honesty when possible.*
   3. *If you begin to fall asleep, I may ask you to stand up.*
5. Introduce paper response option.
   1. *Some groups of questions will have the same response options. When that happens, I will give you a piece of paper to refer to.*
6. Go to REDCap questionnaire on computer and complete questionnaires. See the YETI Data Collection Manual for detailed data collection protocol.
7. Complete REDCap randomization module
   1. Randomization of participants into either the (1) intervention or (2) control arms of the study will take place directly after the baseline survey is completed at Study Visit 1. The randomization module will be pre-programmed into the study’s RedCap just below the questionnaires. The randomization module will assign the participant:
      1. 1 = intervention arm
      2. 0 = control arm
   2. Note: We are **NOT** able to tell the participant which group they are randomized into.
8. Thank for time and inquire about questions.
   1. *Okay we are all done with the questions. Let's take a break and I will come back with your hep C results.*
9. No matter what arm, proceed to break and then disclosure.

Randomize Complete Disclosure

- If the intervention arm, proceed to Intervention Session 1
- If control, proceed to scheduling
- Depending on randomization, staff should fill out the exit form after disclosure or after Session1.
- Upload a picture of the study map onto REDCap or enter manually.

### Schedule Next Visit

1. Schedule next visit in one week using the REDCap calendar.
2. Fill in a Visit Reminder Card and give it to the participant.
3. Confirm preferred contact methods of reminder.

### Pay

1. Ask participant to wait at the Visit Station.
2. Go to kitchen area and get petty cash payment.
3. Fill out petty cash log.
4. Put cash in envelope.
5. Bring payment out to participant.
6. Ask them to sign their payment receipt on REDCap.

### Wrap up

1. Ensure visit checklist steps are all complete.
2. Gather any supplies participant requests.
3. Walk participant out to the street through the South door.

### Documentation

1. Complete and sign visit checklist
   1. Add any notes to the checklist section.
2. Ensure saved and uploaded:
   1. Questionnaires
   2. Scan Navigation Map
3. Update YETI study ID on REDCap as needed
   1. Add partner’s YETI ID if available
   2. If partner ID is not yet available, return to profile at the end of the day and add their ID. Alternatively, if the partner did not show up at the same session, add at a later date.
      1. The data manager will check this on a weekly basis to ensure that there are no missing partner ID’s.
   3. Generate partnership ID
4. Hard copy file
   1. Visit checklist
   2. Navigation Map
5. Petty cash folder
   1. Completed receipt
6. Update live Study Flow sheet
7. Flip station status sign
8. Update team on availability: break or ready for next participant.

# Visit 2 Protocol

Participant presents at front desk and it is established that they are here for Visit 2.

FRONT DESK

Check-in

1. Look at their REDCap profile to see if they are a INDEX or PARTNER participant.
   1. If INDEX ->
      1. Gather “Visit 2 INDEX Packet” with paperclip from top to bottom:
         1. Visit 2 INDEX checklist
         2. Petty cash receipt
         3. Visit reminder
   2. If PARTNER ->
      1. Gather “Visit 2 PARTNER Packet” with paperclip from top to bottom:
         1. Visit 2 PARTNER checklist
         2. Petty cash receipt
         3. Visit reminder
2. Ask if they are with their study partner.
   1. *Are you here with your study partner?*
   2. Circle their response on their checklist
3. Fill in rest of Visit 2 check list using their REDCap profile:
   1. ID
   2. Today’s date
   3. Arm
4. Ask participant to wait in the entry area for interviewer to come grab them
   1. During this time they can grab food and/or pick up harm reduction supplies
5. Message group chat to notify team of a Visit 2 Check in:
6. Update Flow Management software
7. Update their REDCap profile to reflect that they presented for Visit 2

INTERVIEWER

Visit Preparation

1. Review information on their Visit Checklist
2. Review REDCap profile and Visit 1 notes in “Profile”
   1. Quickly check their partners REDCap profile for any important notes
   2. Check if partner YETI ID is missing
      1. If missing, look for partner profile, get their YETI ID, and add to the original participant’s REDCap.
      2. If missing and partner profile doesn’t exist, wait until partner has a REDCap account and add YETI study ID when available.
3. Note their study arm:
   1. Control -> proceed to next step
   2. Intervention ->
      1. Get a copy of their Navigation Map from Hard Copy Box
      2. Prepare any supplies (pens, etc)
      3. Check to see if they are with their partner today

### Welcome

1. Welcome back INDEX participant and introduce self
   1. Welcome back! My name is and I am going to be with you for the next hour and a half again. How are you doing today?
2. Walk participant back to interview station and ask them to get comfortable
3. Put up “Visit in Progress” sign and update “Study Site Flow Sheet”
4. Share what will happen at the visit
   1. To start off, just like last time, I will walk you through what to expect during today’s visit. Does that sound okay?
5. Introduce visit procedures
   1. I will now take a moment to review study procedures for about 5 minutes and answer any of your questions. Do you have any questions before we start?

### Intervention Session (intervention arm only)

1. Begin recording session.
2. Follow intervention Session 2 steps and documentation plan
3. Stop recording session.

### Administer Questionnaires

1. Follow guidance in Best Practices for Survey Administration
2. Before starting, verify that the following are present.
   1. Privacy
      1. Dyad partner not in earshot
      2. White noise machine
      3. Dividers
   2. Comfort and safety
      1. Participant is settled and ready to participate
3. Tell the participant what is about to happen.
   1. Next we will complete the questionnaire. This is expected to last 30 minutes.
4. Remind of ground rules, if needed.
   1. As a reminder, you take a break, go to the bathroom, and stop for food or water at any time.
   2. You can decline any question, but we appreciate your honesty when possible.
   3. If you begin to fall asleep, I may ask you to stand up.
5. Introduce paper response option.
   1. As you will remember from your last visit, some groups of questions will have the same response options. When that happens, I will give you a piece of paper to refer to.
6. Go to REDCap questionnaire on computer. Surveys available will depend on INDEX/PARTNER status and intervention arm.
7. Ensure survey submitted.
8. Thank for time and inquire about questions.
   1. *Okay we are all done. Thank you for your time! Do you have any questions for me before you get you paid and schedule your next visit?*

### Schedule Next Visit

1. Schedule next visit using the RedCap calendar
2. Fill in a Visit Reminder Card and give it to the participant
3. Ask if they have any contact information updates
4. Ask about their preferred for of reminder

### Pay and scan Navigation Map

1. Ask participant to wait at the Visit Station
2. Go to kitchen area and get petty cash payment
3. Scan Navigation Map and make two-sided copy
4. Fill out petty cash log
5. Put cash in envelope
6. Bring payment out to participant
7. Ask them to sign their payment receipt

### Wrap up

1. Ensure visit checklist steps are all complete.
2. Gather any supplies participant requests.
3. Walk participant out to the street through the South door.

**Documentation**

1. Complete and sign visit checklist
   1. Add any notes to the checklist section.
2. Ensure saved and uploaded:
   1. Audio recording
   2. Questionnaires
   3. Navigation Map scan
3. Update REDCap profile
   1. Any notes from visit about the participant, if needed
   2. Partner Info: Confirm if partner YETI ID is available and add to profile for INDEX and PARTNER.
4. Hard copy file
   1. Visit checklist
   2. Navigation Map
5. Petty cash folder
   1. Completed receipt
6. Update live Study Flow sheet
7. Flip station status sign

# Visit 3 Protocol

Participant presents at front desk and it is established that they are here for Visit 3 following the Front Desk Decision Tree.

FRONT DESK

Check-in

1. Gather “Visit 3 Packet” with paperclip from top to bottom:
   1. Visit 3 checklist
   2. Petty cash receipt
   3. Visit reminder
2. Ask if they are with their study partner.
   1. *Are you here with your study partner?*
   2. Circle their response on their checklist
3. Fill in rest of Visit 3 check list using their RedCap profile:
   1. ID
   2. Today’s date
   3. Arm
4. Ask participant to wait in the entry area for interviewer to come grab them
   1. During this time they can grab food and/or pick up harm reduction supplies
5. Message group chat to notify team of a Visit 3 Check in: a.
6. Update Live Study Site Flow Sheet
7. Update their RedCap profile to reflect that they presented for Visit 3

INTERVIEWER

Visit Preparation

1. Review information on their Visit Checklist
2. Review RedCap profile and Visit 1 & 2 notes
3. Quickly check their partners RedCap profile for any important not

**Welcome**

1. Welcome back participant and introduce self
   1. Welcome back! My name is and I am going to be with you for the next hour. How are you doing today?
2. Walk participant back to interview station and ask them to get comfortable
3. Put up “Visit in Progress” sign and update “Study Site Flow Sheet”
4. Share what will happen at the visit
   1. To start off, just like last time, I will walk you through what to expect during today’s visit. Do you have any questions before we start?
   2. *Today’s visit only entails a questionnaire, updates on you contact information, and payment of X dollars.*

### Administer Questionnaires

1. Follow guidance in Best Practices for Survey Administration
2. Before starting, verify that the following are present.
   1. Privacy
      1. Dyad partner not in earshot
      2. White noise machine
      3. Dividers
   2. Comfort and safety
      1. Participant is settled and ready to participate
3. Tell the participant what is about to happen.
   1. Next we will complete the questionnaire. This is expected to last 30 minutes.
4. Remind of ground rules, if needed.
   1. As a reminder, you take a break, go to the bathroom, and stop for food or water at any time.
   2. You can decline any question, but we appreciate your honesty when possible.
   3. If you begin to fall asleep, I may ask you to stand up.
5. Introduce paper response option.
   1. As you will remember from your last visit, some groups of questions will have the same response options. When that happens, I will give you a piece of paper to refer to.
6. Go to RedCap questionnaire on iPad.
7. Ensure survey submitted.
8. Thank for time and inquire about questions.
   1. *Okay we are all done. Thank you for your time! Do you have any questions for me before you get you paid and schedule your next visit?*

### Schedule Next Visit

1. Schedule next visit using the RedCap calendar
2. Fill in a Visit Reminder Card and give it to the participant
3. Ask if they have any contact information updates
4. Ask about their preferred for of reminder

### Pay and scan Navigation Map

1. Ask participant to wait at the Visit Station.
2. Go to kitchen area and get petty cash payment.
3. Fill out petty cash log:
   1. Date
   2. Amount
   3. Participant ID
   4. Staff initials
4. Put cash in an envelope.
5. Bring envelope out to participant at the interview station.
6. Ask them to sign their payment receipt.

### Wrap up

1. Ensure visit checklist steps are all complete.
2. Gather any supplies participant requests.
3. Walk participant out to the street through the South door.

### Documentation

1. Complete and sign visit checklist
   1. Add any notes to the checklist section.
2. Ensure saved and uploaded:
   1. Questionnaires
3. Update RedCap profile
   1. Any notes from visit about the participant, if needed
   2. Partner Info: Confirm if partner YETI ID is available and add to profile for INDEX and PARTNER.
4. Hard copy file
   1. Visit checklist
5. Petty cash folder
   1. Completed receipt
6. Update live Study Flow sheet
7. Flip station status sign

# Visit 4 Protocol

Participant presents at front desk and it is established that they are here for Visit 4 following the Front Desk Decision Tree.

FRONT DESK

Check-in

1. Gather “Visit 4 Packet” with paperclip from top to bottom:
   1. Visit 3 checklist
   2. Petty cash receipt
   3. End of study sheet?
2. Ask if they are with their study partner.
   1. *Are you here with your study partner?*
   2. Circle their response on their checklist
3. Fill in rest of Visit 4 check list using their RedCap profile:
   1. ID
   2. Today’s date
4. Ask participant to wait in the entry area for interviewer to come grab them
   1. During this time they can grab food and/or pick up harm reduction supplies
5. Message group chat to notify team of a Visit 4 Check in: a.
6. Update Live Study Site Flow Sheet
7. Update their RedCap profile to reflect that they presented for Visit 4

INTERVIEWER

Visit Preparation

1. Review information on their Visit Checklist
2. Review RedCap profile and Visits 1-3 notes
3. Quickly check their partners RedCap profile for any important notes

### Welcome

1. Welcome back participant and introduce self
   1. Welcome back! My name is and I am going to be with you for the next hour. How are you doing today?
2. Walk participant back to interview station and ask them to get comfortable
3. Put up “Visit in Progress” sign and update “Study Site Flow Sheet”
4. Share what will happen at the visit
   1. *Today’s visit only entails a questionnaire, updates on you contact information, and payment of fifty dollars.* Do you have any questions before we start?

### Administer Questionnaires

1. Follow guidance in Best Practices for Survey Administration
2. Before starting, verify that the following are present.
   1. Privacy
      1. Dyad partner not in earshot
      2. White noise machine
      3. Dividers
   2. Comfort and safety
      1. Participant is settled and ready to participate
3. Tell the participant what is about to happen.
   1. Next we will complete the questionnaire. This is expected to last 30 minutes.
4. Remind of ground rules, if needed.
   1. As a reminder, you take a break, go to the bathroom, and stop for food or water at any time.
   2. You can decline any question, but we appreciate your honesty when possible.
   3. If you begin to fall asleep, I may ask you to stand up.
5. Introduce paper response option.
   1. As you will remember from your last visit, some groups of questions will have the same response options. When that happens, I will give you a piece of paper to refer to.
6. Go to RedCap questionnaire on iPad.
7. Ensure survey submitted.
8. Thank for time and inquire about questions.
   1. *Okay we are all done. Thank you for your time! Do you have any questions for me before you get you paid and schedule your next visit?*

### Pay

1. Ask participant to wait at the Visit Station.
2. Go to kitchen area and get petty cash payment.
3. Fill out petty cash log:
   1. Date
   2. Amount
   3. Participant ID
   4. Staff initials
4. Put cash in an envelope.
5. Grab study gear
6. Bring envelope and gear out to participant at the interview station.
7. Ask them to sign their payment receipt.

### Wrap up

1. Ensure visit checklist steps are all complete.
2. Gather any supplies participant requests.
3. Walk participant out to the street through the South door.

### Documentation

1. Complete and sign visit checklist
   1. Add any notes to the checklist section.
2. Ensure saved and uploaded:
   1. Questionnaires
3. Update RedCap profile
   1. Any notes from visit about the participant, if needed
   2. Partner Info: Confirm if partner YETI ID is available and add to profile for INDEX and PARTNER.
4. Hard copy file
   1. Visit checklist
5. Petty cash folder
   1. Completed receipt
6. Update live Study Flow sheet
7. Flip station status sig

# VISIT 5 PROCEDURES

Participant presents at front desk and it is established that they are here for Visit 5 following the Front Desk Decision Tree.

FRONT DESK

Check-in

1. Gather “Visit 5 Packet” with paperclip from top to bottom:
   1. Visit 5 checklist
   2. Petty cash receipt
   3. End of study sheet
2. Ask if they are with their study partner.
   1. *Are you here with your study partner?*
   2. Circle their response on their checklist
3. Fill in rest of Visit 5 check list using their REDCap profile:
   1. ID
   2. Today’s date
4. Ask participant to wait in the entry area for interviewer to come grab them
   1. During this time they can grab food and/or pick up harm reduction supplies
5. Message group chat to notify team of a Visit 5 Check in.
6. Update their RedCap profile to reflect that they presented for Visit 5

INTERVIEWER

Visit Preparation

1. Review information on their Visit Checklist
2. Review REDCap profile and Visits 1-4 notes
3. Quickly check their partners RedCap profile for any important notes

**Welcome**

1. Welcome back participant and introduce self
   1. *Welcome back! My name is ____ and I am going to be with you for the next hour or two. How are you doing today?*
2. Walk participant back to interview station and ask them to get comfortable
3. Share what will happen at the visit
   1. *Today is your last official visit with us! It will involve a questionnaire, another short questionnaire about your experiences with the study. The interview will be an opportunity for you to talk more open-endedly about your time working with the YETI study.  Are you willing to answer a few additional questions about your experiences with the study?*

**Administer Questionnaires**

1. Follow guidance in Data Collection Manual
2. Before starting, verify that the following are present.
   1. Privacy
      1. Dyad partner not in earshot
      2. White noise machine
      3. Dividers
   2. Comfort and safety
      1. Participant is settled and ready to participate
3. Tell the participant what is about to happen.
   1. *Next, we will complete the questionnaire. This is expected to last ~45 minutes.*
4. Remind of ground rules, if needed.
5. *As a reminder, you take a break, go to the bathroom, and stop for food or water at any time.*
6. *You can decline any question, but we appreciate your honesty when possible.*
7. *If you begin to fall asleep, I may ask you to stand up.*
8. Introduce paper response option.
9. *As you will remember from your last visit, some groups of questions will have the same response options. When that happens, I will give you a piece of paper to refer to.*
10. Go to RedCap questionnaire and complete.
11. Ensure survey submitted.
12. Thank for time.
13. *Thank you for your participation in this study and for agreeing to participate in this questionnaire about your experiences with the YETI study. This interview/questionnaire will take about 5-10 minutes. Your feedback is important to us and will help improve future research. Please take a few moments to reflect on your experiences participating in the study and provide honest responses to the following questions.*
14. Go to and complete RedCap exit module.
15. Thank for time and inquire about questions.
16. *Okay we are all done! We wanted to remind you that the study is still running for another three years. During that time, we’ll continue to collect information from other participants, and we’ll continue to collect the data from when you go to the doctor. We’ll be analyzing the questionnaires you’ve done and any other data from your doctor’s visits and sharing those results. Thank you for your time! Do you have any questions for me before you get you paid?*

**Pay**

1. Ask participant to wait at the Visit Station.
2. Go to kitchen area and get petty cash payment.
3. Fill out petty cash log:
   1. Date
   2. Amount
   3. Participant ID
   4. Staff initials
4. Put cash in an envelope.
5. Grab study gear
6. Bring envelope and gear out to participant at the interview station.
7. Ask them to sign their payment receipt.

**Wrap up**

1. Ensure visit checklist steps are all complete.
2. Gather any supplies participant requests.
3. Hand participant an exit packet of information and token of appreciation
   1. Exit packet should include:
      1. Harm reduction resources
      2. HCV resources (3 fold flyer)
      3. Testing sites
      4. Flyers for YETI study to distribute to others
4. Walk participant out to the street through the South door.

**Documentation**

1. Complete and sign visit checklist
   1. Add any notes to the checklist section.
2. Ensure saved and uploaded:
   1. Questionnaires
3. Update RedCap profile
   1. Any notes from visit about the participant, if needed
